# AS1411 Nucleolin-Specific Binding Aptamers Reduce Pathological Angiogenesis through Inhibition of Nucleolin Phosphorylation

**DOI:** 10.3390/ijms222313150

**Published:** 2021-12-05

**Authors:** Emilio Iturriaga-Goyon, Oscar Vivanco-Rojas, Fátima Sofía Magaña-Guerrero, Beatriz Buentello-Volante, Ilse Castro-Salas, José Eduardo Aguayo-Flores, Isabel Gracia-Mora, Marisol Rivera-Huerta, Francisco Sánchez-Bartés, Yonathan Garfias

**Affiliations:** 1 MD/Ph.D. (PECEM) Program, Facultad de Medicina, Universidad Nacional Autónoma de México, Ciudad de Mexico 04510, Mexico; iturriemilio@gmail.com; 2 Research Unit, Institute of Ophthalmology, Conde de Valenciana, Chimalpopoca 14, Ciudad de Mexico 06800, Mexico; oscarv@bq.unam.mx (O.V.-R.); fatima.magana@institutodeoftalmologia.org (F.S.M.-G.); bbuentello@institutodeoftalmologia.org (B.B.-V.); ilsecastrosalas@gmail.com (I.C.-S.); dreduardo_aguayo@hotmail.com (J.E.A.-F.); 3 Unidad de Experimentación Preclínica, Department of Inorganic and Nuclear Chemistry, Faculty of Chemistry, Universidad Nacional Autónoma de México, Avenida Universidad 3000, Ciudad de Mexico 04510, Mexico; isabel.gracia@gmail.com (I.G.-M.); river.sunn@gmail.com (M.R.-H.); francisco.bartez@gmail.com (F.S.-B.); 4 Department of Biochemistry, Faculty of Medicine, Universidad Nacional Autónoma de México, Avenida Universidad 3000, Ciudad de Mexico 04510, Mexico

**Keywords:** nucleolin, AS1411, proliferative retinopathy, HUVEC, oxygen induced retinopathy, aortic rings assay, miR-221 expression levels

## Abstract

Proliferative retinopathies produces an irreversible type of blindness affecting working age and pediatric population of industrialized countries. Despite the good results of anti-VEGF therapy, intraocular and systemic complications are often associated after its intravitreal use, hence novel therapeutic approaches are needed. The aim of the present study is to test the effect of the AS1411, an antiangiogenic nucleolin-binding aptamer, using in vivo, ex vivo and in vitro models of angiogenesis and propose a mechanistic insight. Our results showed that AS1411 significantly inhibited retinal neovascularization in the oxygen induced retinopathy (OIR) in vivo model, as well as inhibited branch formation in the rat aortic ex vivo assay, and, significantly reduced proliferation, cell migration and tube formation in the HUVEC in vitro model. Importantly, phosphorylated NCL protein was significantly abolished in HUVEC in the presence of AS1411 without affecting NFκB phosphorylation and -21 and 221-angiomiRs, suggesting that the antiangiogenic properties of this molecule are partially mediated by a down regulation in NCL phosphorylation. In sum, this new research further supports the NCL role in the molecular etiology of pathological angiogenesis and identifies AS1411 as a novel anti-angiogenic treatment.

## 1. Introduction

Pathological angiogenesis in the retina resulting from proliferative diabetic retinopathy (PDR), wet age-related macular degeneration (AMD), retinal artery occlusion, retinal vein occlusion and retinopathy of prematurity (ROP) have in common the production of proangiogenic growth factors [1]. Since the pathophysiology of the aforementioned diseases involves a disruption of the blood-retinal barrier (BRB) and pathological angiogenesis, the final consequence is irreversible blindness affecting working age and pediatric population of industrialized countries. This abnormal and disproportionate hyper-vascularization is a compensatory mechanism to overcome the metabolic equilibrium to the hypoxic retina due to microvessel degeneration [2]. Vascular endothelial growth factor (VEGF) has being strongly implicated in the disruption of the (BRB) [3], perpetuating inflammation [4] and developing proliferative retinopathies [5]. Therefore, intravitreal humanized monoclonal antibodies (huMAb) against VEGF, such as ranibizumab and aflibercept, have being used for PRD, AMD, ROP, macular edema after retinal vein occlusion, vitreous hemorrhage, neovascular glaucoma, among other retinovascular diseases [6]. Unfortunately, VEGF is necessary in many physiologic processes such as wound healing [7], placental trophoblast invasion [8], endometrial regeneration [9], collateral blood vessels formation after myocardial infarction [10], neurogenesis and it has neuroprotective activities [11]. VEGF is produced by different types of cells in the retina, including vascular endothelial cells, pericytes, Müller cells [12], retinal pigment epithelium (RPE) [13] and astrocytes [14]. VEGF has been shown to be critical for ocular development, and acts as a survival factor for the RPE and in non-pathological endothelial cells [15]. Chronic VEGF starvation leads to a significant loss of retinal ganglion cells in patients with AMD [16]. Also, VEGF inhibition has been associated with irreversible photoreceptor damage and RPE-choriocapillaris degeneration [17], endophthalmitis [18], and intraocular pressure elevation [19]. Rare side events after intravitreal injection are traction retinal detachment [20], massive subretinal hemorrhage [21], hemorrhagic macular infarction [22], and retinal vascular occlusions [23]. Although, huMAb against VEGF are applied intravitreal, there is evidence supporting their systemic absorption [24]. Unilateral bevacizumab injections resulted in bilateral response in patients with diabetic macular edema [25]. A prospective non-comparative study, demonstrated that a unique intravitreal injection of bevacizumab was enough to produce a transient narrowing effect on retinal arteriolar diameter in the fellow non-treated eye, with no other possible cause explaining this adverse effect [26]. Due to its chronicity, patients normally receive multiple intravitreal injections of anti-VEGF molecules over time. Many studies found that patients with AMD produce systemic neutralizing antibodies (nAbs) against ranibizumab, arguing that this could explain tachyphylaxis [27]. Tachyphylaxis has been documented in many clinical trials in patients with DR [28,29]. Patients with DR often have other comorbidities such as heart, brain and kidney ischemia [30]. Chronic therapy with VEGF antagonists may prevent the formation of vascular collateral branches and compromise irrigation, hence deteriorating the cardiovascular system. Systemic VEGF inhibition is likely to cause cardiovascular complications [31] and renal dysfunction [32]. 

Since VEGF-VEGFR pathway is ubiquitously present in human organs and tissues, and its function is diverse, it is crucial to find other molecular targets to treat pathological angiogenesis such as DR. VEGF has mitogenic functions in different cells [33], autocrine effects on endothelial cells that plays an important role on survival and retinal cell viability [15], and functions as a chemotactic factor [34]. Chronic inhibition of VEGF could be deleterious and probably has been underestimated [35]. Previous studies have indicated that VEGF stimulation increases cell surface nucleolin (NCL) expression in human microvascular endothelial cells. Importantly, cell surface NCL and VEGF are upregulated in proliferative retinopathies and different types of cancer [36].

NCL is a protein that was firstly described as a nucleolar protein related with ribosome biogenesis, but it has been currently demonstrated that is present in different cell compartments where adopts diverse functions. An altered expression of NCL has been observed in many cancers. For example, in breast and colorectal cancer, NCL protein expression level is increased by six-fold. Previous studies have shown that VEGF, through the VEGFR pathway, leads to phosphorylation of NCL and this event promotes NCL translocation to the membrane. Moreover, NCL redistribution is related to the phosphorylation states of NCL [37]. High cytoplasmic amount of NCL is associated with poor prognosis in patients with gastric and pancreatic cancer [38,39]. The presence of glycosylated form of NCL localized at the surface of glioblastoma cells increases the malignancy of the tumor [40]. Surface and cytoplasmic NCL are differentiated from nuclear NCL by a minor change in their isoelectric point, which could be due to post-translation modifications of surface/ cytoplasmic NCL, such as phosphorylation, which it has been associated to its membrane localization and essential to its angiogenic activities [41]. Cumulative evidence has been proposed cell surface NCL as an important target to inhibit both tumor and angiogenic capacities on various angiogenic-mediated diseases (discussed on [42]). Cell surface NCL has been reported in endothelial and cancer cells acting as a membrane-anchored receptor. At this cellular localization, NCL regulates adhesion [36], differentiation, proliferation and angiogenesis by facilitating the uptake, trafficking and signaling of angiogenic molecules, thus its inhibition promotes affective anti-angiogenesis activities [43]. In this context, our laboratory documented for the first time that NCL is able to translocate onto the de-novo endothelial cell surface after an angiogenic stimulus in an in vivo corneal neovascularization model, and this translocation was abolished when bevacizumab was topically applied, suggesting a direct effect of VEGF in NCL cell surface translocation in aberrant corneal neovessels [44]. Moreover, we have recently described that topical application of nucleolin-binding aptamers AS1411, formerly known as AGRO100, inhibit VEGF-mediated corneal angiogenesis downregulating miR-21 and -221 proangiogenic miRNAs [45]. Aptamers are short single-stranded oligonucleotides that have biological properties that overcome monoclonal antibodies. They have unlimited targets, short production time, cost-effective, thermally stable, high level of tissue penetration and are non-immunogenic (reviewed in [46]). In the present work we describe cell surface NCL as a specific target to reduce pathological angiogenesis by means of using nucleolin-binding aptamers AS1411. 

## 2. Results

### 2.1. Hyperoxia Developed Pathological Angiogenesis in Retinas from Neonatal Mice

**Figure 1:**
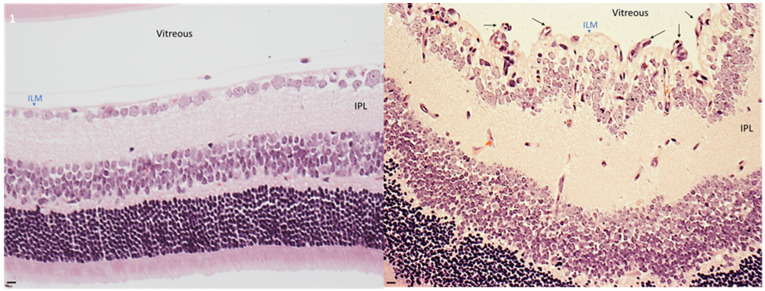
Hyperoxia exposure induces pathological angiogenesis generating a proliferative retinopathy model. Micrographs of retinal histological slides using H&E staining. (**1**) Normoxia exposure developed a physiological angiogenesis in pups at P18. Retinal morphology was normal, no pathological angiogenesis was observed. (**2**) Hyperoxia condition developed pathological angiogenesis located at the superficial retina with a disorganized distribution. Black arrows indicate the neovascular formation at the inner limiting membrane. Representative micrographs of 2 independent assays (*n* = 5 of each assay). Bars = 20 μm.

To determine the differences between mice exposed to normoxia and hyperoxia, neonatal mice were divided aleatory into two groups. Mice exposed to normoxia was assigned to group 1, while mice exposed to hyperoxia was assigned to group 2. As shown in Figure 1, hyperoxia induced severe effects on retinal architecture. Retinas from this group presented a disorganized morphology specially at the internal limiting membrane (ILM), where multiple vessels were present. The pathological angiogenesis was primarily located at the superficial retina as previously described [47]. In contrast, normoxia group had a normal retinal and vascular morphology, corresponding to physiological angiogenesis. Normoxia group had lower vessels and presented a normal distribution through the retinal tissue.

### 2.2. Intravitreal Injection of AS1411 Aptamers Reduced Pathological Angiogenesis in the Oxygen Induced Retinopathy (OIR) Mouse Model

**Figure 2:**
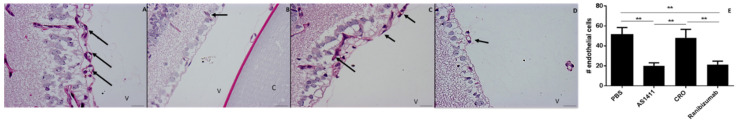
AS1411 reduces retinal angiogenesis in a proliferative retinopathy model. Micrographs of retinal histological slides using PAS staining (40×). Endothelial basement membranes were visualized by positive PAS staining. (**A**) PBS treated retinas developed the same amounts of neovascularization as the CRO group (black arrows). (**B**) AS1411 aptamer presented lower number of neovascular formation at the ILM and the retinal layers architecture was conserved. (**C**) CRO group presented similar amounts of neovascular as the PBS group. (**D**) Ranibizumab treated retinas developed lower number of neovascularization similar to AS1411 treated retinas. (**E**) The bars represent the mean and (±SE) of the endothelial cells per field. PBS and CRO groups had up to around 50 endothelial cells per field; while the mean number of AS1411 and Ranibizumab decreased around to 20 cells per field (a decreased of 2.5-fold compared to PBS and CRO groups). There was no difference between AS1411 aptamer and ranibizumab. Micrographs of 2 independent assays (*n* = 5 of each assay). Bars = 20 µm. ** *p* < 0.01.

To determine the effect of the nucleolin binding aptamers AS1411 on pathological retinal neovascularization in an OIR model, the hyperoxia group was subdivided into four groups. Mice exposed to hyperoxia (A, B, C and D groups) were intravitreally injected from P14 to P17 with different treatments. Group A received 0.01 M PBS; group B received 0.2 nmol AS1411; group C received 0.2 nmol CRO; and group D received 20.8 µM ranibizumab. At P18 all groups were ethically euthanised and eye sections were obtained as described in methods section. The retinal sections were used to visualized endothelial cells extending beyond the ILM into the vitreous. To compare the differences among groups (A, B, C and D) endothelial cell nuclei was quantified. Nuclei counting was achieved by an independent (blinded to the research protocol) veterinary pathologist expert in the mouse OIR model. It is important to consider that mice at P18 have residual fragments of the hyaloid artery located behind the lens, the endothelial nuclei quantification was performed without considering endothelial cells located posterior to the lens. PBS and CRO group presented endothelial cell agglomerations. AS1411 and ranibizumab presented lower amount of endothelial cell agglomerations and normal retinal morphology was visualized. When mice were treated with both PBS and CRO, the number of retinal endothelial cells were similar among them; however, and as expected, when the mice were ranibizumab treated, the endothelial cell number was significantly lower when compared to both PBS and CRO groups. Interestingly, AS1411 was also able to significantly inhibit retinal angiogenesis similar as the ranibizumab group. Neovessels were identified with PAS staining due to the pink coloration that endothelial basement membrane adopts after staining. Neovascular agglomeration was located at the superficial retina, presenting disorganized vessel distribution at the superficial retina. PBS and CRO groups presented abnormal neovessels formation, their distribution was disorganized and protruded into the vitreous. AS1411 and ranibizumab had lower neovessels at the superficial retina, and their distribution presented an organized pattern. Endothelial cell nuclei have an oval shape with central distribution over the cell, this characteristic was useful to count endothelial cell nuclei and determined the effect of the different treatments. The optic nerve was used as a topographic reference to perform the analysis with similar topographic orientation (Figure 2). 

**Figure 3:**
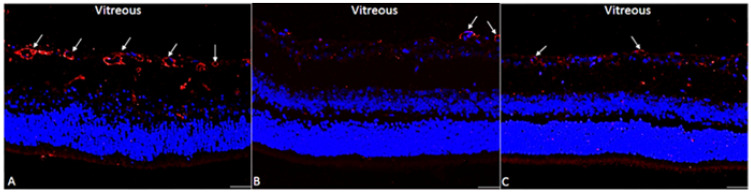
Intravitreally injected reduced Col4-endothelial cell staining at the superficial mice retina. Retinal immunofluorescent micrographs showing Col4 immunolabeling in red. (**A**) CRO intravitreally injected failed to reduce pathological angiogenesis at the retina, these retinas presented augmented number of endothelial cells at the retinal surface, the sprouting neovessels were localized at the vitreous infiltrating through the ILM (white arrows). Extra-retinal neovascularization was characterized by neovessels protruding to the vitreous similar to PDR. (**B**) AS1411 reduced Col4 immunolabeling signal at the retina, hence AS1411 reduced the number of endothelial cells in the retinal ILM near to the vitreous. (**C**) Similarly, to AS1411, ranibizumab treatment reduced immunolabeling signal of Col4-endothelial cells, indicating lower pathological angiogenesis at retinal surface in comparison to the inactive CRO aptamer treatment. These are representative micrographs of 5 independent assays. Nucleus were DAPI (Blue) stained. Bar = 20 µm.

To corroborate the presence of endothelial cell at the ILM, immunolabeling of type IV collagen (Col4) was evaluated by indirect immunofluorescence. Col4 is the main collagen component of the basement membranes, hence is a reliably method to detect endothelial cells in retinal tissue. Our results showed that mice treated with AS1411 presented lower Col4 immunostaining in the retina than CRO treated group. Thus, AS1411 was capable to reduce pathological angiogenesis in the OIR model. Similar to AS1411, ranibizumab presented lower Col4 immunostaining in the retina, and no difference where visualized compared to AS1411 group. In contrast, CRO, failed to inhibit retinal neovascularization. The positive staining for Col4 was mainly observed at the ILM similar to PDR. (Figure 3)

### 2.3. AS1411 Reduces Angiogenesis in Rat Aortic Ring Assay

**Figure 4:**
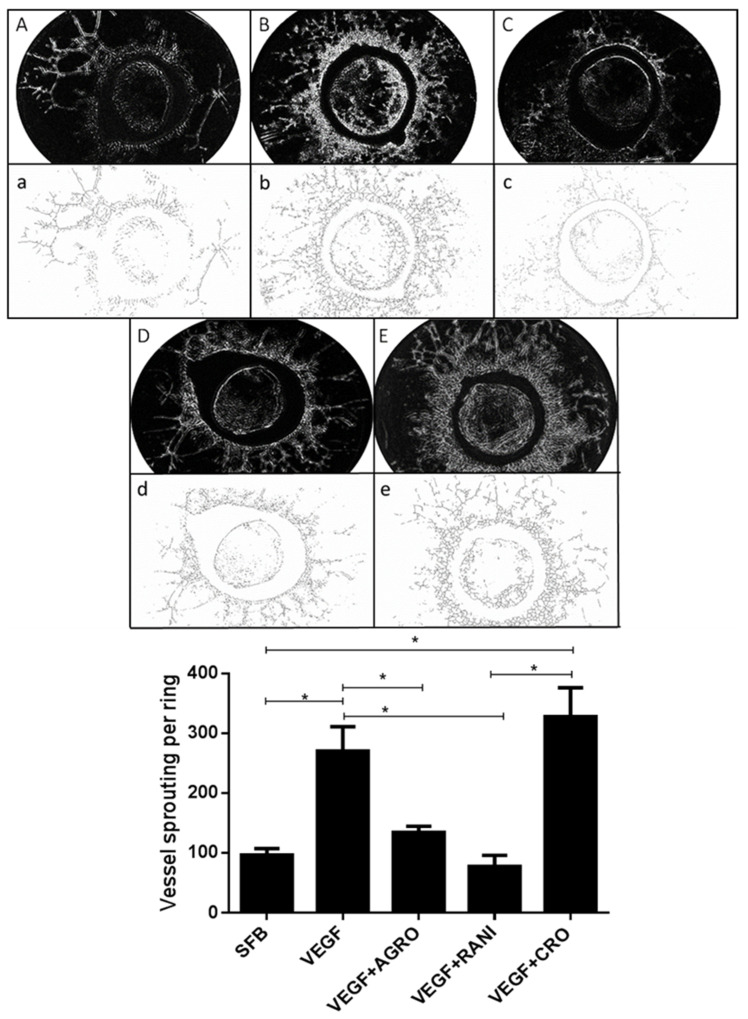
AS1411 reduced vascular sprouting in the rat aortic ring assay. Top panel. Rat aortic rings were cultured for 7 days in matrigel without rhVEGF (**A**,a) or with rhVEGF (**B**,b–**E**,e) in matrigel. The sprouting vessels were visualized and photographed in an inverted microscope, the photographs were converted to 8-bit images (Capital letters) and analyzed and skeletonized using WEKA software (lower-case letters). Control group (**A**,a); rhVEGF alone group (**B**,b); rhVEGF+AS1411 group (**C**,c); rhVEGF+ranibizumab (**D**,d) and rhVEGF+ CRO (**E**,e). Vascular neoformations extended predominantly in a radial shape outwards the aortic rings, besides vascular neoformations extended inwards aortic rings in a minor proportion. Representative images of 15 rings per condition. Bottom panel. The vessels sprouting per aortic ring were compared among the 5 groups. Bars represent the mean (±SE). *n* = 15 rings per condition. * *p* < 0.05.

Rat aortic ring model is an ex vivo assay that has been used to study the inhibitory effect of multiple antiangiogenic molecules. Therefore, we performed this suitable study to determine the antiangiogenic activities of AS1411. As expected, neovessels protruded from the rat aortic rings seeded on matrigel matrix in a time dependent manner, reaching its peak at 7 days after rhVEGF-treatment. Aortic rings stimulated with rhVEGF induced more angiogenesis compared to the control (FBS alone). Endothelial cells in aortic ring are quiescent and unable to spontaneously induced vessel formation. Our results show that *rh*VEGF potentiated vascular sprouting similar to previous studies [48]. Trainable WEKA 3.0 free software (https://imagej.net/plugins/tws/, accessed on 10 May 2021) was used to distinguish neovessels from fibroblast and subtract noisy background, this recognition pattern algorithm confers robust quantification [49] Our results showed that AS1411 significantly reduced vascular vessel sprouting from aortic rings (*p* < 0.05) in comparison with aortic rings exposed to CRO or rhVEGF. Likewise, ranibizumab significantly reduced vascular vessel sprouting compared with both CRO and rhVEGF groups but was similar to AS1411 group (*p* > 0.05). CRO failed to produce any effect on vascular neoformation because this molecule has no biological target in endothelial cells, and as expected, the number of vascular branches was similarly to that treated with rhVEGF. Our results indicated that rat aortic rings exposed to AS1411 produced a smaller number of endothelial branches compared to rings exposed to CRO. Moreover, ranibizumab, produced smaller number of branches compared to rhVEGF and CRO conditions (*p* < 0.05). Suggesting that AS1411 and ranibizumab possess similar antiangiogenic activity in the rat aortic ring assay (Figure 4).

### 2.4. AS1411 Reduced Cell Proliferation, Migration and Tube Formation on Human Umbilical Endothelial Cells (HUVEC)

**Figure 5:**
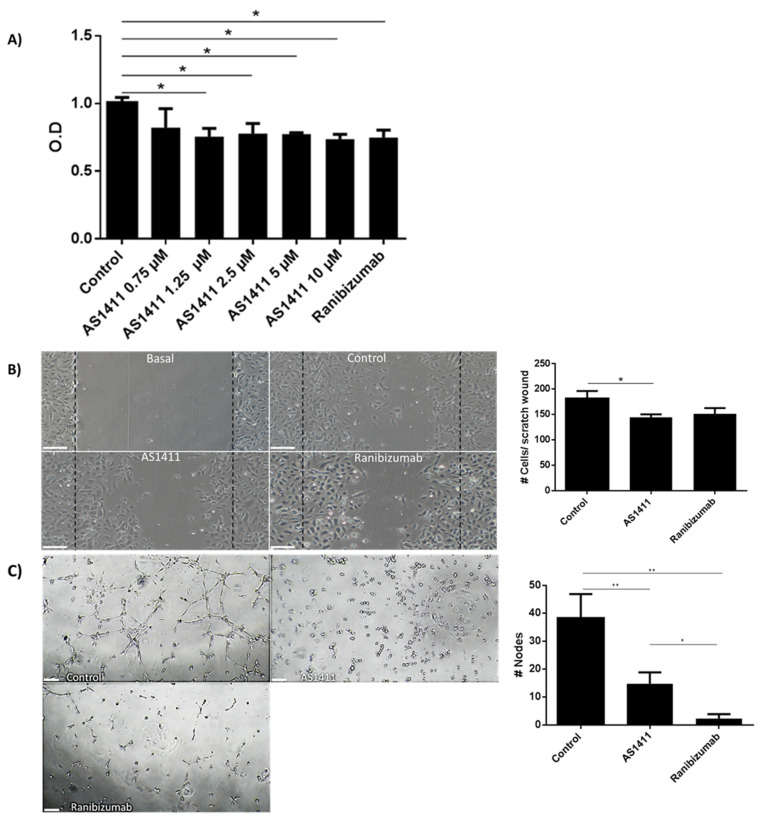
AS1411 reduced HUVEC proliferation, migration and tube formation. (**A**) HUVEC proliferation assay. AS1411 nucleolin-binding aptamers (1.25–10 µM) significantly inhibited HUVEC proliferation. These effects were not dose dependent. As expected ranibizumab inhibited HUVEC proliferation due to VEGF dependent effects over HUVEC. (**B**) HUVEC migration was performed by means of an in vitro scratch assay. Control group was able to significantly induce HUVEC migration due to the growth factors present in the F-12K medium. In contrast, AS1411 (10 µM) were able to significantly reduced HUVEC migration induced by F-12K medium. Similarly, ranibizumab was able to significantly reduced HUVEC migration induced by F-12K. Graphical representation demonstrating that both AS1411 (10 µM) and ranibizumab were able to significantly reduced HUVEC migration in HUVEC. (**C**) HUVEC tube formation assay. Tube formation was significantly inhibited by AS1411 (10 µM) and ranibizumab in comparison to the control group. Bars represent mean (± SE) of 3 independent assays. * *p* < 0.05 and ** *p* <0.01.

It has been widely described that many functions of HUVEC such as proliferation migration and tube formation are VEGF-mediated, thus, antiangiogenic molecules are currently tested on these cells. Firstly, we determined that 1.25 to 10 µM of AS1411 inhibited HUVEC proliferation at 72 h; these results were comparable with those obtained with ranibizumab. (Figure 5A). In confluent HUVEC culture a scratch was performed, and after 24 h photographs were taken in an inverted microscope. The photographs were 8-bit converted and analyzed using ImageJ free software (https://imagej.nih.gov/, accesed on 15 June 2021). Cell migration was considered as the cells that migrated into the basal scratch. Thus, the higher the area, the higher the cell migration. AS1411 and ranibizumab in HUVEC culture, similarly reduced the area covered by cells in the scratch, in comparison with the HUVEC cultured only in complete medium (Figure 5B). Similarly, AS1411 and ranibizumab were equally effective to inhibit HUVEC tube formation on matrigel, in comparison with HUVEC cultured in complete medium (Figure 5C).

### 2.5. AS1411 Inhibited Nucleolin Phosphorylation in HUVEC

**Figure 6:**
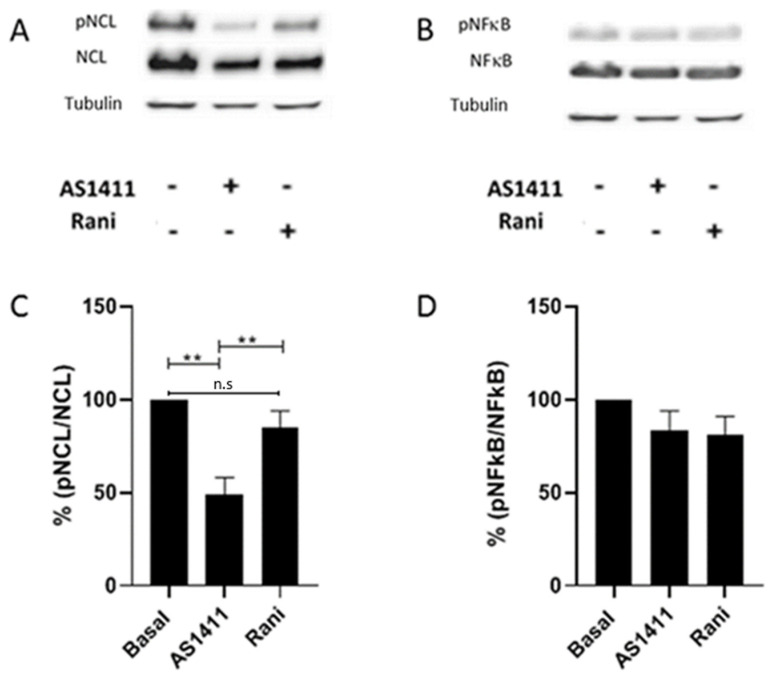
AS1411 decreases phospho-NCL in HUVEC. Cells were cultured in complete medium, AS1411 and ranibizumab were added for 24 h. After incubation period, the cells were lysed and proteins were blotted as indicated. AS1411 (10 µM) was able to significantly inhibit NCL phosphorylation, compared to the control, while ranibizumab did not significantly affect NCL phosphorylation compared to the control (**A**). AS1411 and ranibizumab treatments did not affect NFκB phosphorylation with respect to the control (**B**). Representative blots of three independent assays. Graphical representation of percentage of the pNCL/NCL (**C**), and pNFκB/NFκB ratio (**D**) among groups. Blots are representative of three independent assays. Bars represent mean (±SE). *n* = 3. ** *p* < 0.01.

It has been described that posttranslational modifications such as glycosylation and phosphorylation lead to NCL localization in the cell membrane and mediate its angiogenic activity. We sought to determine whether AS1411 treatment affected NCL phosphorylation in HUVEC. Cells were cultured in the presence of AS1411 or ranibizumab for 24 h, the proteins were obtained and SDS-PAGE electrophoresed and immunoblotted with p-NCL, NCL, p-NFκB, NFκB and tubulin (as a loading control), comparisons were performed with HUVEC cultured in complete medium. Densitometric assays were achieved on the blots. AS1411 nucleolin binding aptamers were able to significantly inhibit the phosphorylation of NCL in HUVEC when compared with HUVEC in the presence of complete medium alone and with ranibizumab. In contrast, ranibizumab did not exert any effect on nucleolin phosphorylation. Interestingly, AS1411 or ranibizumab did not affect the phosphorylation process of NFκB. Suggesting that AS1411 phosphorylation inhibition is exclusively to NCL (Figure 6). 

### 2.6. AS14111 Does Not Affect miR-21 and -221 Expression in HUVEC

**Figure 7:**
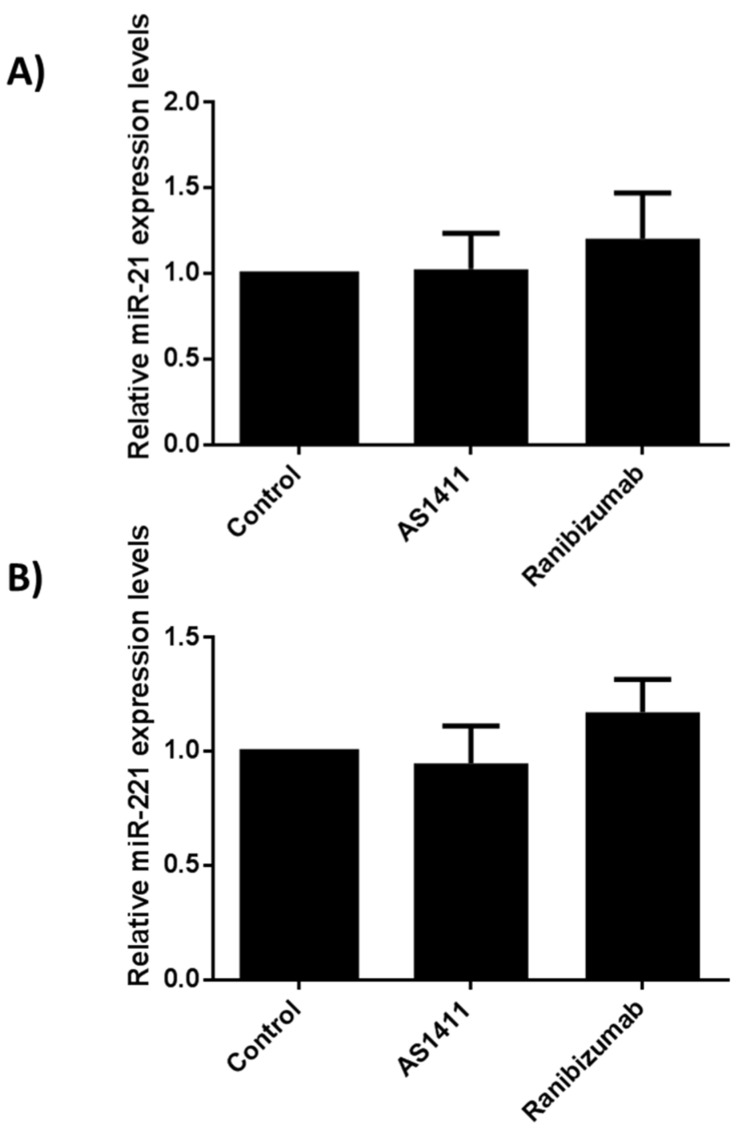
AS14111 aptamer treatment does not affect miR-21 and -221 relative expression levels in HUVEC. (**A**) AS1411 aptamer and ranibizumab failed to reduced miR21 relative expression levels in cultured HUVEC. (**B**) AS1411 aptamer and ranibizumab failed to reduced miR-221 relative expression levels in cultured HUVEC. The bars represent the mean (±SE) of three independent assays.

As aforementioned, AS1411 nucleolin binding aptamers were capable to inhibit the expression of both miR-21 and -221 in corneal cells stimulated with rhVEGF. In an attempt to determine whether AS1411 exerted similar effects on HUVEC, total RNA was obtained, and miR-21 and -221 expressions were compared among conditions. The expression of miR-21 and -221 angiomiRs from both AS1411 and ranibizumab treatments were similar and there were no statistical differences when compared with the non-treated cells, suggesting that AS1411 nucleolin-binding aptamers exert their antiangiogenic activities in HUVEC independently form angiomiRs (Figure 7).

## 3. Discussion

In the present study we describe that nucleolin binding AS1411 aptamers were able to reduce pathological angiogenesis in the OIR in vivo model, reduced aortic branching in the rat aortic ring ex vivo model of angiogenesis, and reduced proliferation, cell migration and tube formation in the HUVEC in vitro model. Mechanistically, we showed that AS1411 aptamers exerted their anti-angiogenic activities through inhibition of NCL phosphorylation and downregulation of the expression of the angiogenic miR-221 molecule. We tested AS1411 in three different experimental models which mimic PDR. Using OIR in vivo model and rat aortic ring ex vivo model, we showed that AS1411 induced an inhibition in neoangiogenesis similarly as ranibizumab did, which is a VEGF-inhibitor clinically approved for human retinopathies. Although anti-VEGF treatments are widely used with good results in patients, recent studies report that long-term treatments with these drugs induce several complications such as retinal atrophy development [50]. Furthermore, repeated intravitreal injections of anti-VEGF molecules cause the activation of fibrocytes and development of fibrovascular membrane, leading to tractional retinal detachment and retinal hemorrhage [51]. Interestingly, in our experiments we did not observe any toxic effect with the AS1411 intravitreal injection, suggesting its safety profile. Moreover, AS1411 aptamer has shown promising utility as a treatment for cancers in Phase I and Phase II clinical trials without causing major side effects [52]. The aim of this study arose from the fact that NCL is a nucleolar protein involved in cell growth, survival and tumorigenesis. Previous studies in our group revealed that there is a cell surface localization of NCL in angiogenic cells in a suture-induced corneal neovascularization model [44] and that AS411 was able to inhibit corneal neovascularization downregulating the angiogenic miR-21 and-221 molecules [45]. Our results are consistent with previous studies where intravitreal injection or topical application of G-Quartet aptamer targeting NCL ameliorated choroidal neovascularization in a model of age-related macular degeneration [53]. Recently, proteomic analysis of OIR-treated retinas and samples obtained from human PDR patients showed that the vascular leakage positively correlates with the amount of MyH9, a heavy chain of nonmuscle myosin IIA [54]. Myh9 regulates angiogenesis through signal transductions modulating VEGF-A expression in tissue [55] and by promoting cell surface NCL translocation [36]. The low increase in NCL expression demonstrated by mass spectrometry in the OIR model, suggests that the effects of NCL in angiogenesis are related to its translocation from the nucleus to the cell surface rather than its de novo synthesis [54]. Recently, N6L nucleolin-binding pseudo peptide intraperitoneally administered significantly diminished retinal neoangiogenesis in two models of proliferative retinopathy, reinforcing the rationale to use cell surface NCL as a possible target in proliferative retinopathies [56]. To determine the effect of AS1411 on retinal vasculature we used eye cross sections to count endothelial cell nuclei in retinas in the OIR model. This method has been validated to evaluate retinal neoangiogenesis in the ROP in vivo model [57]. AS1411 intravitreally injected diminished the number of endothelial nuclei in PAS stain sections, which was corroborated using immunostaining for Col4. It has been described that cell surface NCL is responsible of driving the angiogenesis process [36,58]. Therefore, inhibiting cell surface NCL with AS1411 inhibits nucleolin-dependent angiogenesis in the OIR in vivo model. We consider that although these results showed an effect of AS1411 on retinal neovascular angiogenesis, further studies in whole flat mount retinas need to be evaluated to determine the impact of AS1411 on vaso-obliteration, neovascular tuft formation, neovessels regrowth and tuft regression [59].

In our experiments aortic rings treated with rhVEGF presented more tube formations compared to the aortic rings incubated with FBS alone, in accordance to a previous study [60]. After 7 days of culture, the vascular proliferation induced with rhVEGF was significantly inhibited with AS411, but not with the inactive oligonucleotide CRO. This suggest that the inhibition was mediated specifically through cell-surface NCL. As expected, ranibizumab significantly reduced the number of vascular branches in the aortic rings compared to aortic rings cultured with rhVEGF alone. This is the first study describing the antiangiogenic effects of AS1411 on both the OIR in vivo model and the rat aortic angiogenesis ex vivo assays. The antiangiogenic activity of AS1411 was also corroborated in HUVEC. HUVEC are VEGF-dependent cells. Thus, they are suitable cells to test antiangiogenic molecules and to dissect some mechanistic insights. AS1411 inhibited essential functions of HUVEC such as proliferation, migration and tube formation. HUVEC constitutively express cell surface NCL which serves as a receptor for kallistatin and the interaction of both cell surface NCL and kallistatin mediates the antiangiogenic effects of kallistatin [61]; similarly, AS1411 inhibited HUVEC proliferation. MyH9 a protein associated with cell surface NCL promotes angiogenesis favoring cell motility [62], whether AS1411 nucleolin-binding aptamers downregulate MyH9 expression in HUVEC cannot be ruled out. It has been described that cell surface antagonists such as the pseudopeptide HB-19 and N6L inhibit the in vitro adhesion, migration and HUVEC proliferation through SRC, ERK1/2, AKT and FAK kinases [63,64]. Although VEGF can promote the activation of downstream genes such as protein kinases (e.g., PKC, Akt), and thus could regulate the distribution of NCL by the PI3K/Akt pathway and lead to phosphorylation of NCL. Similarly, in the present study we show that AS1411 aptamers were able to inhibit HUVEC proliferation and tube formation. These cell activities were closely related to the inhibition of NCL phosphorylation. In this context, NCL phosphorylation is associated with its cell surface translocation [37], while downregulating NCL phosphorylation, significantly inhibits cell migration [41,65]. In order to determine whether these findings were mediated through the down regulation of NCL phosphorylation, we evaluated the ratio of phopho-NCL/NCL. Our findings indicate that AS1411 significantly reduced phopho-NCL/NCL ratio protein levels [61]. However, the effect of AS1411 on protein kinases down regulation or protein phosphatases up regulation is still matter of further research. 

In contrast, ranibizumab failed to reduce phospho-NCL/NCL ratio protein levels. Thus, AS1411 mechanisms of action over proliferation, cell migration and tube formation on HUVEC are different by those mediated by the antiangiogenic molecule ranibizumab. Surprisingly, AS1411 did not affect the phosphorylation of NFκB; these results disagree with those reported by Girva et al., showing that AGRO-100 aptamers (same sequence as AS1411) inhibited NFκB activity in tumor cells suggesting a role in regulation of IKK complex [66]; but, NFκB signaling is often deregulated in many cancer cells, which could explain our findings. miRNAs are small non-coding RNAs that function as a negative regulators of gene expression. Numerous types of miRNAs have been related with the angiogenic process, defined as angiomiRs [67]. There are angiomiRs that regulate angiogenesis, such as miR-221 and miR-222 [68]. For example, mir-21 has been implicated as an angiogenic inducer through the activation of AKT and ERK pathway. Meanwhile, it has been described that mir-221 modulates the expression of multiple targets through VEGF signaling output [69]. Although, it has been described an intricate relationship between phospo-NCL and miRNA maturation in cardiomiocytes [70], miR-21 and -221 expression in HUVEC remained unmodified in the presence of AS411 and ranibizumab in the present study. Suggesting that AS1411 nucleolin-binding antiangiogenic activities on HUVEC are angiomiR-21 and-221 independent.

## 4. Materials and Methods

### 4.1. Reagents

The oligodeoxynucleotides AS1411 (5′-dGGTGGTGGTGGTTGTGGTGGTG GTGG -3′) and the inactive control oligonucleotide CRO (5′-dCCTCCT CCTCCTTCTCCTCCTCCTCC-3′), were purchased from IDT (Coralville, IA). We use ranibizumab as a negative control for all angiogenesis assays, as previously described. Ranibizumab (Lucentis) was obtained from Novartis (Mexico City, Mexico), and stored at 2–8 °C [71] (Montassar et al., 2017). The other reagents were purchased from Sigma-Aldrich (St. Louis, MO), unless otherwise stated. 

### 4.2. Animal Model

The study was performed in accordance with the ARVO statement for the use of animals in Ophthalmology and Vision Research as well as with the Mexican Federal Regulations for use of laboratory animals (NOM-062-200-1999). The protocol was approved by the Comité Interno para el Cuidado y Uso de los Animales de Laboratorio–Facultad de Química, UNAM (FQ/CICUAL/335/18; 9 October 2018). The OIR mouse model was performed as previously described. Briefly, 7-day-old (p7) C57BL/6J (*n* = 20) were exposed to 75% oxygen for 5 days, this caused a down-regulation of VEGF and therefore vaso-obliteration, afterwards the mice returned to normoxia to induce ischemic retinopathy characterized by pathological neovascularization that can be seen as vascular neoformation with disorganized morphology and at P17–P18 [72]. It is well known that retinal neovascularization is maximal at p17 and has been associated with high concentration of VEGF after ischemia. To compare physiological retinal neovascularization, the normoxia group was exposed to 21% oxygen during 17 days, at P18 normoxia group was properly euthanized to obtain the ocular globes. All groups were housed and bred at 22 °C ± 2 °C with 40–70% humidity and at a 12:12 light/dark cycle with access to a standard rodent pellets and water *ad libitum*. Nursing dams stayed with their own litters throughout the entire experiment in order to nourish the mice pups. The smallest pups according to their body weight on P14 were excluded from the experiment.

### 4.3. Intravitreal Injection of Nucleolin-Binding Aptamer and Controls

C57BL/6 mice (2 weeks old, 6–7.5 g) were used. All mice at P14 were anaesthetized using sevoflurane (Abbott Laboratories, Mexico City, Mexico), and treated with one drop of tetracaine, tropicamide and 2.5% phenylephrine (Sophia, Guadalajara, Mexico) to reduce corneal reflex and dilate pupils, respectively. To determine the effect of AS1411 in the OIR mice model, mice exposed to hyperoxia were randomly divided into four different groups and injected with 0.5 µL (final volume) using a Hamilton syringe depending of the group: (A) group of OIR treated with 0.01 M sterile PBS as a positive control of angiogenesis or as a negative control of treatment, (B) group of OIR treated with 0.2 nmol sterile AS1411 as previously reported by Leaderer et al. [53], (C) group of OIR treated with 0.2 nmol sterile CRO which is the negative control for AS1411 due to its changes in citosines instead of guanines, and (D) group treated with 20.8 µM sterile ranibizumab corresponding to a negative control of angiogenesis. The procedure of intravitreal injection was performed using a 10 µL gastight Hamilton syringe equipped with a 33-gauge sharp needle under a stereoscopic microscope (Carl Zeiss, Jena, Germany). The incision was made with a sharp needle 1 mm posterior to the temporal limbus. After the intravitreal injections were performed, topical ophthalmic antibiotic was applied. Intravitreal injections were performed for 4 consecutive days starting on P14. 

### 4.4. Quantification of Neovascular Proliferative Retinopathy

At P18 mice were ethically euthanized administrating 60 mg kg-1pentobarbital (Pisabental, PiSA, Guadalajara, Mexico) by intraperitoneal injection. Immediately, eyes specimens were carefully enucleated under a stereoscopic microscope (Carl Zeiss, Jena, Germany) and immersed in 4% paraformaldehyde solution (Bio Basic, Ontario, Canada) during 24 h. Tissue was properly dehydrated by gradually replacing water with alcohol and thereafter xylene. Eyes were placed in warm paraffin wax for 30 min to achieve proper embedding. Serial sections of 6 µm were obtained in a sagittal orientation. The mice cornea was oriented in a parallel way to the optic nerve; and then mounted on microscope glass slides. To achieve tissue staining all slides were deparaffinized and rehydrated with xylene/ethanol decrease gradient concentration. Periodic Acid-Schiff (PAS) and hematoxylin-eosin (H&E) staining was performed. Nuclei from new vessels could be distinguished from other structures in the retina, only nuclei which protrude through the inner limiting membrane (ILM) into the vitreous body. This method quantifies extra-retinal neovascularization abnormally present in a vasoproliferative retinopathy model. Single blinded test was performed for the endothelial nuclei quantification; this was achieved by a certified veterinary pathologist from UNIPREC (preclinical investigation unit that has been certified by COFEPRIS [Mexican Federal Commission for Protection against Sanitary Risks]). Cross-sections that included the optic nerve were excluded because normal vessels emerged from the optic nerve. 

### 4.5. Immunofluorescence

Eye tissues cuts on pre-charged slides were deparaffinized and rehydrated with a gradient of xylene/ethanol concentrations. Antigen retrieval was performed by heating the samples in 10 mM citrate buffer (pH 6.0). Samples were incubated for 1 h with blocking solution (BSA 5% + Triton 0.1%). Sections were incubated with rabbit polyclonal antibodies to type IV Collagen (ab6586) overnight at 4 °C, afterwards, the samples were incubated with Alexa Fluor 594 donkey anti-rabbit for 1 h, at room temperature. Negative controls were performed by leaving out the primary antibody. Finally, the samples were washed, mounted with DAPI and images were obtained with an ApoTome II microscope (Carl Zeiss, Jena, Germany). 

### 4.6. Rat Aortic Ring Assay

Rat aortic rings were cultured on a matrix and supplemented with recombinant human (rh)VEGF (R&D Systems, Minneapolis, MN, USA) to stimulate angiogenesis. Quantifying sprouting vessels from rat aortic ring have been used to determine the effect of antiangiogenic molecules. The aortic ring assay was performed as previously described. Briefly, male wistar rats (*n* = 4, 200 (±10) g, 6–7 weeks old) were purchased from the National Autonomous University of Mexico (UNAM) and housed in a room with a 12-h light dark cycle maintained at 22 (±2 °C), with a relative humidity of 60 ± 2%. Food and water were supplied *ad libitum*. Then, 96-well plates were covered with 70 µL growth factors reduced-(GFR) Matrigel (BD Biosciences, San Jose, CA, USA) at 4 °C. Animals were ethically euthanised, thoracic aortas were dissected and fibroadipose tissue was carefully removed, aortic tissue was cut sagittally in order to obtain 1 mm rings [73]. Aortic rings were placed on GFR-Matrigel and cultured with endothelial basal medium (Clonetics Lonza, Basel, Switzerland), supplemented with 2.5% fetal bovine serum (FBS) and antibiotics. Aortic rings were randomly distributed into five groups: (1) Only with 2.5% FBS to evaluate basal vessels sprouting, (2) 2.5% FBS plus 30 ng/mL rhVEGF, (3) 2.5% FBS plus 30 ng/mL rhVEGF supplemented with 150 µM AS1411 since rat aortic ring is a tissue structure aptamer dose was inferred by previous reports using higher aptamer concentration because is an explant tissue [74,75], (4) 2.5% FBS plus 30 ng/mL rhVEGF supplemented with CRO 150 µM used as an aptamer control, (5) 2.5% FBS plus 30 ng/mL rhVEGF supplemented with 20.8 mM ranibizumab. Aortic rings were incubated 7 days in aseptically conditions at 37 °C with 5% CO_2_. Aortic ring cultures were visualized and photographed under an inverted microscope, and a digital image was generated for later analysis. Images were analyzed by manually encircling the outgrowth area and computing square pixels. All images were analyzed by trainable Weka segmentation (version 3.0). Aortic ring skeletonization was analyzed using FIJI software (https://imagej.nih.gov, accesed on 15 June 2021).

### 4.7. Aptamer Effects on Human Umbilical Vein Endothelial Cells (HUVEC)

Human umbilical vein endothelium cells were gently gifted by Dr. Arturo Flores from the Instituto Nacional de Perinatología México, City, Mexico; obtained from umbilical cords of healthy fetuses from uncomplicated pregnancies and healthy women that underwent cesarean sections at term with no evidence of hypertension disorders through the pregnancy in order to avoid endothelial cell damage. HUVECs were cultured in Kaighn’s modification of Ham’s F12 medium (F-12K) purchased from Invitrogen (Carlsbad, CA, USA) supplemented with 20% fetal bovine serum (FBS), endothelial cell growth supplement (ECGS) and 100 µg/mL heparin. HUVEC were successively seeded until confluent monolayer was reached. HUVEC were divided aleatory in three groups: (1) complete F12K medium was assigned as control group, (2) complete F12K medium supplemented with 10 µM AS1411 aptamer as previously reported by Futami et al., [76,77] and (3) complete F12K medium supplemented with 20.8 mM ranibizumab. Proliferation, cell migration and tube formation assays were performed using the same AS1411 and ranibizumab concentrations. Proliferation evaluation: proliferations assays were achieved using MTT (3-[4,5-dimethylthiazol-2-yl]-2,5 diphenyl tetrazolium bromide) in 96-well cell culture plates at a density of 2 × 10^3^ cells per well. Briefly, at the end of the cell culture (72 h), HUVEC were treated with 20 µL of MTT solution (5 mg/mL) and incubated for 4 h. Then the medium was removed and replaced with 150 µL of 0.01 M sterile PBS. Immediately after the water insoluble formazan crystals were completely dissolved, optical density values were obtained with an automatic spectrophotometer (Multiskan Ascente, Lab X, ON, Canada) using a wavelength of 590 nm. The MTT assays were performed in triplicate. Cell migration evaluation: scratch wound healing assays were performed as previously described with modification [78]. Briefly, HUVEC (2 × 10^4^) were seeded in 6 well cell culture plates. Shaped wounds were performed with a sterile p200 pipet tip across each well, in order to create a cell free area to allow cell migration; to remove loose and dead cells, each well was gently rinsed with FBS-free medium and cultured in different conditions during 24 h. Five images of the scraped area were captured using a phase contrast microscope Olympus CK-2 (Olympus, Southend-on-Sea, UK). The cells that migrated into the basal wounded area at five different points per image were measured. The wound healing assays were performed in triplicate. *Tube formation*: HUVEC (2 × 10^3^) were seeded on 24 well microplates previously coated with 50 µL of GFR-Matrigel and 600 µL of complete F12K medium was added with different treatments. After 3 h of incubation, tube formation was documented using an inverted microscope. Tube formation assays were performed in triplicate.

### 4.8. Nucleolin Immunoblotting

Proteins were isolated from the first elute of the RLT buffer lysis using the RNAeasy Kit (Qiagen, Hilden, Germany), as the manufacturer indicates. Briefly, after the different culture conditions, HUVEC were lysed with RLT buffer (300 μL/1 × 10^6^ cells); afterwards, the lysed cells were passed through a DNA/RNA binding column and the eluted fraction which contains the proteins was incubated with 4-fold the initial volume of iced-cold acetone overnight at –20 °C. The precipitated proteins were washed with ethanol and suspended in 1× Laemmli buffer without β-mercaptoethanol and bromophenol blue. The proteins were quantified by the BCA/DC method using a protein quantification assay kit (BioRad, Hercules, California, CA, USA). Twenty μg of total protein was loaded in each lane; samples were resolved by SDS-Tris-Glycine polyacrylamide gel electrophoresis on precast gradient gels (BioRad, Hercules, CA, USA). Immediately, gels were transferred onto nitrocellulose membrane with 0.45 µm (BioRad, Hercules, CA, USA) in Tris-glycine buffer containing 10% methanol. Proteins were detected by immunoblotting. Membranes were reprobed as described in the corresponding figure legend.

### 4.9. MiRNA Arrays

Total RNA was extracted from HUVEC that were cultured in different conditions using the RNAeasy Kit (Qiagen, Hilden, Germany), as indicated by the manufacturer. One hundred ng of total RNA were reverse transcribed, and then, real-time qPCR assays were performed. The relative expression of miR-21 and mir-221 normalized to that of snRNA U6 was calculated and presented by values of 2 -^ΔΔ^CT (relative expression). The assays were performed in triplicate.

### 4.10. Statistical Analysis

All data were assembled, and Mann-Whitney U tests were used to determine differences between groups in case of non-parametric distribution, using *p* < 0.05 as statistically significant. Statistical analysis was made by GraphPad Prism software (GraphPad, La Jolla, California, CA, USA).

## 5. Conclusions

Aptamers are novel molecules that are being used to improve therapeutic outcomes in humans, with lower side effect than conventional chemotherapy such as myelosuppression and cardiotoxicity. These molecules have higher binding affinity and specificity compared to monoclonal antibodies. The chemical production presents lower costs compared to therapeutic antibodies, and they are not immunogenic. All these properties make them suitable for biomedical applications [46,79]. AS1411 is an aptamer that specifically binds to NCL, it has anti-proliferative effects over tumor cells, inhibits NF-κB pro-survival mechanism, blocks DNA-replication and induces cell cycle arrest. Furthermore, anti-VEGF intravitreal therapy produces tachyphylaxis over time, and systemic side effect have been reported. In this study we have shown that intravitreal application of AS1411 reduces retinal neovascularization in the OIR model, reduces aortic vessel branching in rat aortic rings model. AS1411 also reduced HUVEC proliferation, cell migration and tube formation. These antiangiogenic effects are mediated through inhibition of NCL phosphorylation. These experimental approaches mimic pathological angiogenesis stimulated by VEGF and hypoxia such as DR; hence, the use of these aptamers opens new perspectives to treat retinal neovascularization in humans.

## Data Availability

Data are provided upon request.

## References

[1] Dreyfuss J.L., Giordano R.J., Regatieri C.V. (2015). Ocular Angiogenesis. J. Ophthalmol..

[2] Sapieha P., Hamel D., Shao Z., Rivera J.C., Zaniolo K., Joyal J.S., Chemtob S. (2010). Proliferative retinopathies: Angiogenesis that blinds. Int. J. Biochem. Cell Biol..

[3] Harhaj N.S., Felinski E.A., Wolpert E.B., Sundstrom J.M., Gardner T.W., Antonetti D.A. (2006). VEGF Activation of Protein Kinase C Stimulates Occludin Phosphorylation and Contributes to Endothelial Permeability. Investig. Ophthalmol. Vis. Sci..

[4] Rangasamy S., McGuire P.G., Franco Nitta C., Monickaraj F., Oruganti S.R., Das A. (2014). Chemokine mediated monocyte trafficking into the retina: Role of inflammation in alteration of the blood-retinal barrier in diabetic retinopathy. PLoS ONE.

[5] Liu Y., Shen J., Fortmann S.D., Wang J., Vestweber D., Campochiaro P.A. (2017). Reversible retinal vessel closure from VEGF-induced leukocyte plugging. JCI Insight.

[6] Gross J.G., Glassman A.R., Jampol L.M., Inusah S., Aiello L.P., Antoszyk A.N., Baker C.W., Berger B.B., Bressler N.M., Writing Committee for the Diabetic Retinopathy Clinical Research Network (2015). Panretinal Photocoagulation vs Intravitreous Ranibizumab for Proliferative Diabetic Retinopathy: A Randomized Clinical Trial. JAMA.

[7] Wilgus T.A., Matthies A.M., Radek K.A., Dovi J.V., Burns A.L., Shankar R., DiPietro L.A. (2005). Novel function for vascular endothelial growth factor receptor-1 on epidermal keratinocytes. Am. J. Pathol..

[8] Knöfler M., Pollheimer J. (2013). Human placental trophoblast invasion and differentiation: A particular focus on Wnt signaling. Front. Genet..

[9] Graubert M.D., Ortega M.A., Kessel B., Mortola J.F., Iruela-Arispe M.L. (2001). Vascular repair after menstruation involves regulation of vascular endothelial growth factor-receptor phosphorylation by sFLT-1. Am. J. Pathol..

[10] Zhao T., Zhao W., Chen Y., Ahokas R.A., Sun Y. (2010). Vascular endothelial growth factor (VEGF)-A: Role on cardiac angiogenesis following myocardial infarction. Microvasc. Res..

[11] Wang Y., Kilic E., Kilic Ü., Weber B., Bassetti C.L., Marti H.H., Hermann D.M. (2004). VEGF overexpression induces post-ischaemic neuroprotection, but facilitates haemodynamic steal phenomena. Brain.

[12] Wang J., Xu X., Elliott M.H., Zhu M., Le Y.-Z. (2010). Müller Cell-Derived VEGF Is Essential for Diabetes-Induced Retinal Inflammation and Vascular Leakage. Diabetes.

[13] Saint-Geniez M., Kurihara T., Sekiyama E., Maldonado A.E., Amore P.A. (2009). An essential role for RPE-derived soluble VEGF in the maintenance of the choriocapillaris. Proc. Natl. Acad. Sci. USA.

[14] Scott A., Powner M.B., Gandhi P., Clarkin C., Gutmann D.H., Johnson R.S., Ferrara N., Fruttiger M. (2010). Astrocyte-derived vascular endothelial growth factor stabilizes vessels in the developing retinal vasculature. PLoS ONE.

[15] Alon T., Hemo I., Itin A., Pe’er J., Stone J., Keshet E. (1995). Vascular endothelial growth factor acts as a survival factor for newly formed retinal vessels and has implications for retinopathy of prematurity. Nat. Med..

[16] Beck M., Munk M.R., Ebneter A., Wolf S., Zinkernagel M.S. (2016). Retinal Ganglion Cell Layer Change in Patients Treated With Anti–Vascular Endothelial Growth Factor for Neovascular Age-related Macular Degeneration. Am. J. Ophthalmol..

[17] Julien S., Biesemeier A., Taubitz T., Schraermeyer U. (2014). Different effects of intravitreally injected ranibizumab and aflibercept on retinal and choroidal tissues of monkey eyes. Br. J. Ophthalmol..

[18] Mccannel C.A. (2011). Meta-Analysis of Endophthalmitis after Intravitreal Injection of Anti–Vascular Endothelial Growth Factor Agents: Causative Organisms and Possible Prevention Strategies. RETINA.

[19] Hoang Q.V., Mendonca L.S., Della Torre K.E., Jung J.J., Tsuang A.J., Freund K.B. (2012). Effect on Intraocular Pressure in Patients Receiving Unilateral Intravitreal Anti-Vascular Endothelial Growth Factor Injections. Ophthalmology.

[20] Meyer C.H., Michels S., Rodrigues E.B., Hager A., Mennel S., Schmidt J.C., Helb H.-M., Farah M.E. (2011). Incidence of rhegmatogenous retinal detachments after intravitreal antivascular endothelial factor injections. Acta Ophthalmol..

[21] Karagiannis D.A., Mitropoulos P., Ladas I.D. (2009). Large Subretinal Haemorrhage following Change from Intravitreal Bevacizumab to Ranibizumab. Ophthalmologica.

[22] Kelkar A., Gandhi P., Amoaku W., Kelkar J., Kelkar S., Raut P., Shah R. (2014). Hemorrhagic macular infarction after intravitreal bevacizumab for chronic multifocal central serous chorioretinopathy. Case Rep. Ophthalmol..

[23] Mansour A.M., Bynoe L.A., Welch J.C., Pesavento R., Mahendradas P., Ziemssen F., Pai S.A. (2010). Retinal vascular events after intravitreal bevacizumab. Acta Ophthalmol..

[24] Avery R.L., Castellarin A.A., Steinle N.C., Dhoot D.S., Pieramici D.J., See R., Couvillion S., Nasir M.A.A., Rabena M.D., Le K. (2014). Systemic pharmacokinetics following intravitreal injections of ranibizumab, bevacizumab or aflibercept in patients with neovascular AMD. Br. J. Ophthalmol..

[25] Hanhart J., Tiosano L., Averbukh E., Banin E., Hemo I., Chowers I. (2014). Fellow eye effect of unilateral intravitreal bevacizumab injection in eyes with diabetic macular edema. Eye.

[26] Peyman M., Peyman A., Lansingh V.C., Orandi A., Subrayan V. (2018). Intravitreal bevacizumab versus ranibizumab: Effects on the vessels of the fellow non-treated eye. J. Curr. Ophthalmol..

[27] Rosenfeld P.J., Brown D.M., Heier J.S., Boyer D.S., Kaiser P.K., Chung C.Y., Kim R.Y. (2006). Ranibizumab for Neovascular Age-Related Macular Degeneration. N. Engl. J. Med..

[28] McCloskey C.F., Mongan A.-M., Chetty S., McAteer D.M.J., Quinn S.M. (2018). Aflibercept in Diabetic Macular Oedema Previously Refractory to Standard Intravitreal Therapy: An Irish Retrospective Study. Ophthalmol. Ther..

[29] Eghøj M.S., Sørensen T.L. (2012). Tachyphylaxis during treatment of exudative age-related macular degeneration with ranibizumab. Br. J. Ophthalmol..

[30] Shah K., Gandhi A., Natarajan S. (2018). Diabetic Retinopathy Awareness and Associations with Multiple Comorbidities: Insights from DIAMOND Study. Indian J. Endocrinol. Metab..

[31] Schmid M.K., Bachmann L.M., Fäs L., Kessels A.G., Job O.M., Thiel M.A. (2015). Efficacy and adverse events of aflibercept, ranibizumab and bevacizumab in age-related macular degeneration: A trade-off analysis. Br. J. Ophthalmol..

[32] Tschulakow A., Christner S., Julien S., Ludinsky M., van der Giet M., Schraermeyer U. (2014). Effects of a single intravitreal injection of aflibercept and ranibizumab on glomeruli of monkeys. PLoS ONE.

[33] Yamamoto H., Rundqvist H., Branco C., Johnson R.S. (2016). Autocrine VEGF Isoforms Differentially Regulate Endothelial Cell Behavior. Front. Cell Dev. Biol..

[34] Evans I.M., Kennedy S.A., Paliashvili K., Santra T., Yamaji M., Lovering R.C., Britton G., Frankel P., Kolch W., Zachary I.C. (2017). Vascular Endothelial Growth Factor (VEGF) Promotes Assembly of the p130Cas Interactome to Drive Endothelial Chemotactic Signaling and Angiogenesis. Mol. Cell Proteom..

[35] Hombrebueno J.R., Ali I.H.A., Xu H., Chen M. (2015). Sustained intraocular VEGF neutralization results in retinal neurodegeneration in the Ins2(Akita) diabetic mouse. Sci. Rep..

[36] Huang Y., Shi H., Zhou H., Song X., Yuan S., Luo Y.J.B. (2006). The angiogenic function of nucleolin is mediated by vascular endothelial growth factor and nonmuscle myosin. Blood.

[37] Wu D.M., Zhang P., Liu R.Y., Sang Y.X., Zhou C., Xu G.C., Yang J.L., Tong A.P., Wang C.T. (2014). Phosphorylation and changes in the distribution of nucleolin promote tumor metastasis via the PI3K/Akt pathway in colorectal carcinoma. FEBS Lett..

[38] Gilles M.E., Maione F., Cossutta M., Carpentier G., Caruana L., Di Maria S., Houppe C., Destouches D., Shchors K., Prochasson C. (2016). Nucleolin Targeting Impairs the Progression of Pancreatic Cancer and Promotes the Normalization of Tumor Vasculature. Cancer Res..

[39] Qiu W., Zhou F., Zhang Q., Sun X., Shi X., Liang Y., Wang X., Yue L. (2013). Overexpression of nucleolin and different expression sites both related to the prognosis of gastric cancer. APMIS.

[40] Galzio R., Rosati F., Benedetti E., Cristiano L., Aldi S., Mei S., D’Angelo B., Gentile R., Laurenti G., Cifone M.G. (2012). Glycosilated nucleolin as marker for human gliomas. J. Cell. Biochem..

[41] Huang F., Wu Y., Tan H., Guo T., Zhang K., Li D., Tong Z. (2019). Phosphorylation of nucleolin is indispensable to its involvement in the proliferation and migration of non-small cell lung cancer cells. Oncol. Rep..

[42] Dhez A.C., Benedetti E., Antonosante A., Panella G., Ranieri B., Florio T.M., Cristiano L., Angelucci F., Giansanti F., Di Leandro L. (2018). Targeted therapy of human glioblastoma via delivery of a toxin through a peptide directed to cell surface nucleolin. J. Cell. Physiol..

[43] Ferrara B., Belbekhouche S., Habert D., Houppe C., Vallee B., Bourgoin-Voillard S., Cohen J.L., Cascone I., Courty J. (2021). Cell surface nucleolin as active bait for nanomedicine in cancer therapy: A promising option. Nanotechnology.

[44] Quiroz-Mercado J., Ramírez-Velázquez N., Partido G., Zenteno E., Chávez R., Agundis-Mata C., Jiménez-Martínez M.C., Garfias Y. (2016). Tissue and cellular characterisation of nucleolin in a murine model of corneal angiogenesis. Graefes Arch. Clin. Exp. Ophthalmol..

[45] Vivanco-Rojas O., García-Bermúdez M.Y., Iturriaga-Goyon E., Rebollo W., Buentello-Volante B., Magaña-Guerrero F.S., Bates P., Pérez-Torres A., Garfias Y. (2020). Corneal neovascularization is inhibited with nucleolin-binding aptamer, AS1411. Exp. Eye Res..

[46] Iturriaga-Goyon E., Buentello-Volante B., Magana-Guerrero F.S., Garfias Y. (2021). Future Perspectives of Therapeutic, Diagnostic and Prognostic Aptamers in Eye Pathological Angiogenesis. Cells.

[47] Calzi S.L., Shaw L.C., Moldovan L., Shelley W.C., Qi X., Racette L., Quigley J.L., Fortmann S.D., Boulton M.E., Yoder M.C.J.J.i. (2019). Progenitor cell combination normalizes retinal vascular development in the oxygen-induced retinopathy (OIR) model. JCI Insight.

[48] Zhu W.-H., MacIntyre A., Nicosia R.F. (2002). Regulation of Angiogenesis by Vascular Endothelial Growth Factor and Angiopoietin-1 in the Rat Aorta Model: Distinct Temporal Patterns of Intracellular Signaling Correlate with Induction of Angiogenic Sprouting. Am. J. Pathol..

[49] Arganda-Carreras I., Kaynig V., Rueden C., Eliceiri K.W., Schindelin J., Cardona A., Sebastian Seung H. (2017). Trainable Weka Segmentation: A machine learning tool for microscopy pixel classification. Bioinformatics.

[50] Eng V.A., Rayess N., Nguyen H.V., Leng T. (2020). Complete RPE and outer retinal atrophy in patients receiving anti-VEGF treatment for neovascular age-related macular degeneration. PLoS ONE.

[51] Sato T., Ooto S., Suzuki M., Spaide R.F. (2015). Retinal pigment epithelial tear after intravitreal aflibercept for neovascular age-related macular degeneration. Ophthalmic Surg. Lasers Imaging Retin..

[52] Trinh T.L., Zhu G., Xiao X., Puszyk W., Sefah K., Wu Q., Tan W., Liu C. (2015). A Synthetic Aptamer-Drug Adduct for Targeted Liver Cancer Therapy. PLoS ONE.

[53] Leaderer D., Cashman S.M., Kumar-Singh R. (2015). Topical application of a G-Quartet aptamer targeting nucleolin attenuates choroidal neovascularization in a model of age-related macular degeneration. Exp. Eye Res..

[54] Vähätupa M., Nättinen J., Jylhä A., Aapola U., Kataja M., Kööbi P., Järvinen T.A.H., Uusitalo H., Uusitalo-Järvinen H. (2018). SWATH-MS Proteomic Analysis of Oxygen-Induced Retinopathy Reveals Novel Potential Therapeutic Targets. Investig. Ophthalmol. Vis. Sci..

[55] Morrison A.R., Yarovinsky T.O., Young B.D., Moraes F., Ross T.D., Ceneri N., Zhang J., Zhuang Z.W., Sinusas A.J., Pardi R. (2014). Chemokine-coupled β2 integrin-induced macrophage Rac2-Myosin IIA interaction regulates VEGF-A mRNA stability and arteriogenesis. J. Exp. Med..

[56] Darche M., Cossutta M., Caruana L., Houppe C., Gilles M.-E., Habert D., Guilloneau X., Vignaud L., Paques M., Courty J. (2020). Antagonist of nucleolin, N6L, inhibits neovascularization in mouse models of retinopathies. FASEB J..

[57] Liu D., Xiong S.Q., Shang L., Tian X.F., Yang J., Xia X.B. (2014). Expression of netrin-1 receptors in retina of oxygen-induced retinopathy in mice. BMC Ophthalmol..

[58] Christian S., Pilch J., Akerman M.E., Porkka K., Laakkonen P., Ruoslahti E. (2003). Nucleolin expressed at the cell surface is a marker of endothelial cells in angiogenic blood vessels. J. Cell Biol..

[59] Scott A., Fruttiger M. (2010). Oxygen-induced retinopathy: A model for vascular pathology in the retina. Eye.

[60] Zhu W.-H., Iurlaro M., MacIntyre A., Fogel E., Nicosia R.F. (2003). The Mouse Aorta Model: Influence of Genetic Background and Aging on bFGF- and VEGF-Induced Angiogenic Sprouting. Angiogenesis.

[61] Huang X.P., Wang X., Xie X.L., Zhang G.P., Lv F.J., Weng W.T., Qiu F., Li Z.F., Lin J.S., Diao Y. (2018). Cell surface expression of nucleolin mediates the antiangiogenic and antitumor activities of kallistatin. Oncotarget.

[62] Yang B., Liu H., Bi Y., Cheng C., Li G., Kong P., Zhang L., Shi R., Zhang Y., Zhang R. (2020). MYH9 promotes cell metastasis via inducing Angiogenesis and Epithelial Mesenchymal Transition in Esophageal Squamous Cell Carcinoma. Int. J. Med. Sci..

[63] Birmpas C., Briand J.P., Courty J., Katsoris P. (2012). Nucleolin mediates the antiangiogenesis effect of the pseudopeptide N6L. BMC Cell Biol..

[64] Birmpas C., Briand J.P., Courty J., Katsoris P. (2012). The pseudopeptide HB-19 binds to cell surface nucleolin and inhibits angiogenesis. Vasc. Cell.

[65] Koutsioumpa M., Polytarchou C., Courty J., Zhang Y., Kieffer N., Mikelis C., Skandalis S.S., Hellman U., Iliopoulos D., Papadimitriou E. (2013). Interplay between αvβ3 integrin and nucleolin regulates human endothelial and glioma cell migration. J. Biol. Chem..

[66] Girvan A.C., Teng Y., Casson L.K., Thomas S.D., Jüliger S., Ball M.W., Klein J.B., Pierce W.M., Barve S.S., Bates P.J. (2006). AGRO100 inhibits activation of nuclear factor-kappaB (NF-kappaB) by forming a complex with NF-kappaB essential modulator (NEMO) and nucleolin. Mol. Cancer Ther..

[67] Sabatel C., Malvaux L., Bovy N., Deroanne C., Lambert V., Gonzalez M.-L.A., Colige A., Rakic J.-M., Noël A., Martial J.A. (2011). MicroRNA-21 Exhibits Antiangiogenic Function by Targeting RhoB Expression in Endothelial Cells. PLoS ONE.

[68] Poliseno L., Tuccoli A., Mariani L., Evangelista M., Citti L., Woods K., Mercatanti A., Hammond S., Rainaldi G. (2006). MicroRNAs modulate the angiogenic properties of HUVECs. Blood.

[69] Hu J., Ni S., Cao Y., Zhang T., Wu T., Yin X., Lang Y., Lu H.J.P.O. (2016). The angiogenic effect of microRNA-21 targeting TIMP3 through the regulation of MMP2 and MMP9. PLoS ONE.

[70] Tong Z., Tang Y., Jiang B., Wu Y., Liu Y., Li Y., Xiao X. (2019). Phosphorylation of nucleolin is indispensable to upregulate miR-21 and inhibit apoptosis in cardiomyocytes. J. Cell. Physiol..

[71] Montassar F., Darche M., Blaizot A., Augustin S., Conart J.-B., Millet A., Elayeb M., Sahel J.-A., Goazigo A.R.-L., Sennlaub F. (2017). Lebecetin, a C-type lectin, inhibits choroidal and retinal neovascularization. FASEB J..

[72] Lange C., Ehlken C., Stahl A., Martin G., Hansen L., Agostini H.T. (2009). Kinetics of retinal vaso-obliteration and neovascularisation in the oxygen-induced retinopathy (OIR) mouse model. Graefes Arch. Clin. Exp. Ophthalmol..

[73] Rymo S.F., Gerhardt H., Wolfhagen Sand F., Lang R., Uv A., Betsholtz C. (2011). A Two-Way Communication between Microglial Cells and Angiogenic Sprouts Regulates Angiogenesis in Aortic Ring Cultures. PLoS ONE.

[74] Aravind A., Jeyamohan P., Nair R., Veeranarayanan S., Nagaoka Y., Yoshida Y., Maekawa T., Kumar D.S. (2012). AS1411 aptamer tagged PLGA-lecithin-PEG nanoparticles for tumor cell targeting and drug delivery. Biotechnol. Bioeng..

[75] Taghdisi S.M., Danesh N.M., Ramezani M., Yazdian-Robati R., Abnous K. (2018). A novel AS1411 aptamer-based three-way junction pocket DNA nanostructure loaded with doxorubicin for targeting cancer cells in vitro and in vivo. Mol. Pharm..

[76] Futami K., Kimoto M., Lim Y.W.S., Hirao I. (2019). Genetic Alphabet Expansion Provides Versatile Specificities and Activities of Unnatural-Base DNA Aptamers Targeting Cancer Cells. Mol. Ther.-Nucleic. Acids.

[77] Reyes-Reyes E.M., Šalipur F.R., Shams M., Forsthoefel M.K., Bates P.J. (2015). Mechanistic studies of anticancer aptamer AS1411 reveal a novel role for nucleolin in regulating Rac1 activation. Mol. Oncol..

[78] Liang P., Jiang B., Lv C., Huang X., Sun L., Zhang P., Huang X. (2013). The expression and proangiogenic effect of nucleolin during the recovery of heat-denatured HUVECs. Biochim. Biophys. Acta.

[79] Ali M.H., Elsherbiny M.E., Emara M. (2019). Updates on Aptamer Research. Int. J. Mol. Sci..

